# The molecular and neurochemical basis of altered intrinsic neural timescales in major depressive disorder: a multimodal imaging-transcriptomic investigation of esketamine and sertraline treatment

**DOI:** 10.1186/s12888-026-08477-0

**Published:** 2026-08-05

**Authors:** Xiang Liu, Yuanzhi He, Lifeng Li, Zhanxiang Hu, Haijun Li, Lihao Wu, Liping Zhang, Zihao Su, HengRui Liu, Guojiang Wu, Dechang Peng

**Affiliations:** 1 Jiangxi Provincial Key Laboratory for Precision Pathology and Intelligent Diagnosis, Department of Radiology, The First Affiliated Hospital, Jiangxi Medical College, Nanchang University, Nanchang, 330006 China; 2 Queen Mary School, Jiangxi Medical College, Nanchang University, Nanchang, 330006 China; 3 The School of Medical Imaging, Changsha Medical University, Changsha, 410000 China; 4 Department of Psychosomatic Medicine, The First Affiliated Hospital, Jiangxi Medical College, Nanchang University, Jiangxi, 330006 China; 5 Department of Biochemistry, University of Cambridge, Tennis Court Road, Cambridge, CB2 1QW UK

**Keywords:** Major depressive disorder, Esketamine, Intrinsic neural timescale, Sertraline

## Abstract

**Introduction:**

Major depressive disorder (MDD) is characterized by dysfunction in higher-order cortical regions involved in emotional and cognitive processes; however, its neurobiological basis remains unclear. Pharmacological treatments, including esketamine and sertraline, produce rapid antidepressant effects. We investigated the local intrinsic neural dynamics underlying these antidepressant effects using intrinsic neural timescales (INT), which measure the duration and capacity of information integration in localized brain regions.

**Methods:**

A total of 57 healthy controls and 41 patients with MDD were included. All patients with MDD received six intravenous infusions of esketamine (0.25 mg/kg) and two weeks of sertraline treatment. All participants underwent resting-state fMRI to quantify INT alterations at the voxel and brain network levels, followed by spatial correlation analyses linking autocorrelation metrics of INT signals to transcriptomic data from the Allen Human Brain Atlas and PET-derived neurotransmitter receptor maps. The spatial correlation between PLS2 scores and case–control t-statistic maps did not pass rigorous spin permutation testing (*r* = 0.484, *p* = 0.068) and did not meet the conventional significance threshold of 0.05. Accordingly, all subsequent enrichment analyses based on PLS2 results are presented as exploratory preliminary observations for hypothesis generation.

**Results:**

Compared with healthy controls, patients with MDD exhibited significantly elevated INT levels in the left and right precuneus. Following treatment, patients with MDD showed significantly increased INT in the left occipital midline region and left calcarine cortex. At the brain network level, INT levels were significantly reduced in the default mode network (DMN) in patients with MDD compared with healthy controls. Compared with the pre-treatment state, the cerebellar network (CN) showed significantly elevated INT levels after treatment. Partial least squares regression analysis suggested potential associations between INT alterations and spatial gene expression gradients particularly those associated with immune responses, hormonal regulation, neutrophils, regulatory T cells, and glutamatergic synapses. Cell-type enrichment analysis identified excitatory and inhibitory neurons as key cellular contributors. Alterations in INT also correlated with cortical 5-HT1b and NAT receptor density, suggesting a role for inhibitory neurotransmission in temporal integration deficits.

**Conclusion:**

This study advances understanding of treatment-related brain abnormalities in patients with MDD from the perspective of local neural dynamics. These findings support a multiscale pathophysiological framework involving brain connectivity dynamics, molecular architecture, and neurochemical regulation in MDD.

## Introduction

Major depressive disorder (MDD) is a prevalent mental disorder classified by the World Health Organization as a leading cause of global disability. With a lifetime prevalence of 4.4% in the general population, MDD significantly impairs an individual’s quality of life [1]. The primary clinical features of MDD include persistent low mood, hopelessness, changes in appetite and sleep, fatigue, loss of interest or pleasure in daily activities, and physical symptoms [2]. The diagnosis of MDD primarily relies on the Diagnostic and Statistical Manual of Mental Disorders, Fifth Edition (DSM-5), which requires patients to exhibit five or more symptoms that persist for at least two weeks. Currently, the diagnosis of MDD is largely based on patient symptoms, and clinical manifestations vary substantially among individuals. The heterogeneity of depressive symptoms is associated with multiple factors, including genetics, environment, biology, social psychology, brain anatomy, and alterations in brain function [3].

Current treatments for MDD include medication, psychotherapy, and physical therapy. Medication remains the most common primary treatment strategy [4]. Pharmacological treatments primarily modulate inhibitory neurotransmitters within the central nervous system. Gamma aminobutyric acid (GABA), glutamate (Glu), and monoamine neurotransmitters play crucial pathophysiological roles in mood disorders [5]. In 2019, the U.S. Food and Drug Administration approved esketamine as an antidepressant with a novel mechanism of action [6]. As an N-methyl-d-aspartate receptor antagonist, esketamine rapidly improves mood disturbances and suicidal ideation in patients with MDD by modulating the GABA/glutamatergic system [7]. In recent years, esketamine has been used in adolescent [8]and adult [9]depression populations due to its safety profile and favorable therapeutic outcomes. Sertraline is a selective serotonin reuptake inhibitor (SSRI) that exerts antidepressant effects primarily by regulating monoamine neurotransmitters and has good efficacy and tolerability [10]. Although these two drug classes can improve depressive symptoms and alleviate suicidal ideation in patients with MDD, the neuroanatomical features underlying their therapeutic responses remain unclear. Exploring the neural basis of MDD using neuroimaging techniques can enhance understanding of the potential pathophysiological mechanisms underlying the disorder. This information is crucial for refining diagnostic and treatment strategies.

Functional magnetic resonance imaging (fMRI), which is radiation-free and non-invasive, has advanced the exploration of neural mechanisms in MDD. Common resting-state fMRI techniques include amplitude of low-frequency fluctuation (ALFF) [11], regional homogeneity (ReHo) [12], functional connectivity (FC) [13], and brain network analysis [14]. Lai et al. reported that, compared with the anxiety group, patients with MDD exhibited significantly reduced regional homogeneity in the left cingulate cortex and bilateral anterior cingulate cortex. Changes in ReHo values in the anterior cingulate cortex may represent specific pathophysiological alterations in MDD [15]. An ALFF study confirmed that patients with first-episode adolescent MDD exhibited heightened spontaneous neuronal activity in the medial prefrontal and anterior cingulate cortices, which may explain rumination and impaired attention [16]. A longitudinal independent component analysis study found that a neurodevelopmental model attributed depressive symptoms in adolescent females to abnormalities in functional connectivity patterns among the amygdala, striatum, and prefrontal cortex, which may represent potential targets for early depression detection [17]. These studies have identified regional brain function and network alterations associated with depressive symptoms. Neuroimaging studies examining the relationship between MDD and treatment response have also progressed. One seed-based functional MRI study found that changes in functional connectivity patterns between the cingulate cortex and the whole brain after intravenous ketamine infusion in patients with MDD could predict the antisuicidal effects of ketamine [18]. An FC study found that enhanced FC between the amygdala and medial prefrontal cortex and caudate nucleus may serve as a predictor of the antidepressant effects of ketamine and its efficacy in reducing suicidal ideation [19]. These studies have identified relationships among localized brain function, functional network changes, and antidepressant effects, helping to identify potential therapeutic targets.

The intrinsic neural timescale (INT) improves understanding of local intrinsic brain activity. INT is calculated by assessing the autocorrelation amplitude of resting-state fMRI brain activity to characterize heterogeneity in neural timescales [20]. This measure quantifies intrinsic temporal scales and the time range over which brain regions integrate information [21]. The duration for which neural information is stored across different neural regions is a fundamental functional characteristic of localized brain areas and reflects hierarchical differences between brain structure and neural dynamics [22]. INT is currently used to assess self-awareness and neurodevelopmental status in patients with psychiatric disorders. Watanabe et al. reported that individuals with autism exhibited shorter temporal scales in the sensory/visual cortex and longer temporal scales in the right caudate nucleus, reflecting alterations in higher-order cognitive symptoms in this population [23]. In a study of MDD, reduced intrinsic temporal scales in sensorimotor-, visual-, and cognitive-related regions were associated with disease severity and distinct disease stages [24]. Another study of depression showed that individuals with MDD may exhibit reduced intrinsic temporal scales and spatial disruption in the brain, specifically in the default mode network (DMN) and control networks, offering new insights into the neural basis of depression [25].

Genetic factors play a significant role in brain connectivity, and whole-brain gene expression maps integrate relationships between macroscopic brain networks and microscopic molecular components [26]. The Allen Human Brain Atlas (AHBA) has been used to identify transcriptomes associated with human neuroimaging studies. Integrating multimodal neuroimaging data revealed relationships between the transcriptome and brain function in regions exhibiting neurogenetic vulnerability to MDD [27]. Limited progress has been made in depression research. Li et al. found that gene expression in patients with MDD was correlated with morphologically similar networks, with glial cell- and neuron-specific transcriptional alterations contributing to MDD-specific morphologically similar network associations [28]. Another study found that changes in medial prefrontal cortex thickness in patients with MDD were associated with abnormal functional connectivity patterns. The cortical regions involved in these connectivity patterns showed coupling with AHBA expression, revealing dysregulation of cortical somatostatin-producing interneurons and astrocytes [29].

Based on this, we link the INT associated with MDD to the AHBA to advance our understanding of the relationship between molecular mechanisms and functional alterations in depression. First, we explored MDD-related changes in the intrinsic neural timescale across two independent cohorts. Second, we further investigated the pattern of changes in the intrinsic neural timescale in MDD patients following pharmacotherapy (esketamine combined with sertraline). Finally, we further linked INT abnormalities to anatomical pattern gene expression to explore the relationship between INT and transcriptional patterns, as well as the molecular functions and cell types of INT-associated genes. These observations help us understand how whole-brain gene expression and cell types capture functionally distinct differences in MDD that are molecularly validated.

## Materials and methods

### Subjects

**Fig. 1:**
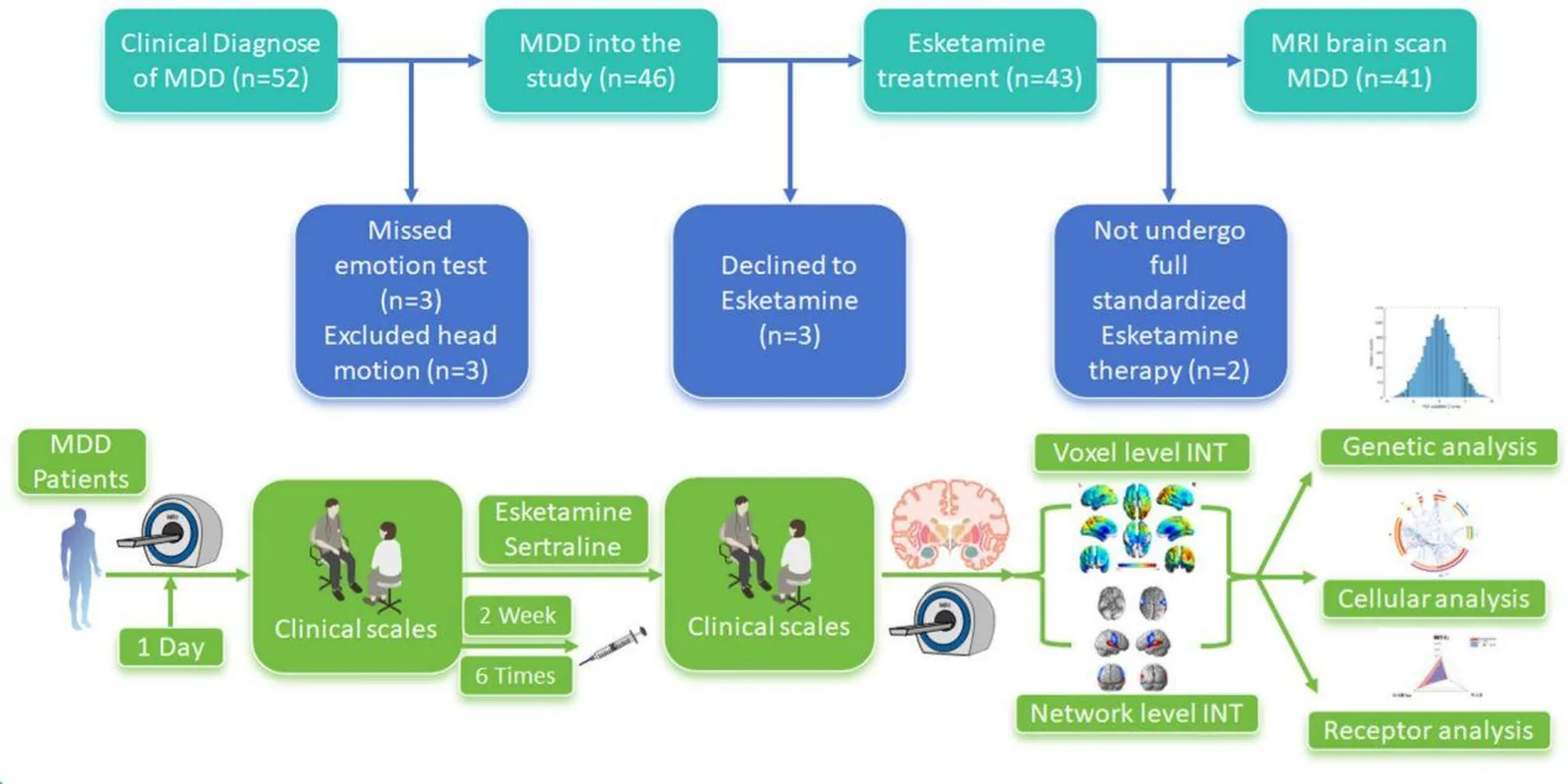
MDD patient collection flowchart

This was a longitudinal study evaluating the combination of esketamine and sertraline over a 2-week period (Fig. 1). The study enrolled 41 patients with major depressive disorder (MDD) and 57 healthy controls. All MDD patients were enrolled at the Department of Psychosomatic Medicine, The First Affiliated Hospital of Nanchang University between June 2022 and March 2025. MDD patient inclusion criteria: Right-handed, aged 14–65 years, no gender restrictions; meets the diagnostic criteria for a major depressive episode in the Diagnostic and Statistical Manual of Mental Disorders, Fifth Edition (DSM-5); MDD defined as a total score ≥ 24 on the 17-item Hamilton Depression Scale (HAMD-17) with a suicide risk item score ≥ 1; Capable of communicating and providing written informed consent; No abnormal signal foci in brain parenchyma on routine MRI scan. This study was approved by the Ethics Committee of the First Affiliated Hospital of Nanchang University (Approval No.: IIT2022130). Informed consent was obtained from all participants prior to the study, and they signed informed consent forms.

Exclusion criteria for MDD patients: (1) Exclude patients with other mental disorders, including organic mental disorders, schizophrenia, schizoaffective disorder, bipolar disorder, etc.; (2) Exclude patients who are currently experiencing unstable physical illnesses, such as cardiovascular disease, diabetes, hypertension, hyperthyroidism, hypothyroidism, etc.; (3) Exclude patients with a recent history of alcohol abuse or illicit drug misuse.; (4) Exclude patients with central nervous system disorders, such as neurodegenerative diseases, epilepsy, dementia, or traumatic brain injury.; (5) Exclude patients with organic lesions detected by routine MRI, such as brain tumors or stroke.; (6) Exclude patients with MRI contraindications, such as pacemaker implantation or claustrophobia.; (7) Exclude patients who have previously experienced ineffective results with esketamine or who have contraindications to esketamine administration.; (8) Exclude patients with a history of electroconvulsive therapy or other NMDA receptor antagonist treatment within the past 6 months.

All subjects underwent a rigorous screening process, including clinical evaluation by psychiatrists, review of medical histories, and laboratory testing. During screening, the research team ensured the exclusion of all potential confounding factors that could interfere with the results, thereby guaranteeing the homogeneity of the final enrolled patients and the validity of the data.

### Drug use

Esketamine: Indications: This product is indicated for use in combination with an oral antidepressant (sertraline) to alleviate depressive symptoms in patients with major depressive disorder who have acute suicidal ideation or behavior. Dosage Form: Injection; Strength: 50 mg. According to the drug instructions, the dosage is 0.25 mg/kg. After calculating the dosage based on the patient’s weight, prepare esketamine in a 1 g/L solution using normal saline. Administer the solution via intravenous infusion using a microinfusion pump at a rate of 1mL/min. The administration frequency is 3 times per week for 2 consecutive weeks. Esketamine Injection Protocol: (1) Fast for 8 h prior to administration; (2) Monitor temperature, blood pressure, pulse, and respiration hourly before treatment; (3) No food or drink during injection and for 30 min afterward; (4) Family members must remain present throughout the injection and for 30 min afterward to ensure patient safety. Concurrently, the experimental group received conventional oral antidepressant therapy (sertraline) but was prohibited from concomitant use of barbiturates or other anesthetic agents. Sertraline dosage: 50 mg/tablet. Dosage regimen: 50 mg once daily on days 1–4, 100 mg once daily on days 5–8, and 150 mg once daily thereafter.

### Clinical scale assessment

Depression and anxiety assessment utilizes the Hamilton Anxiety Scale (HAMA) and Hamilton Depression Scale (HAMD) to evaluate patients’ anxiety and depressive symptoms. The HAMA comprises 14 items (somatic anxiety and psychic anxiety), each rated on a 5-point scale from 0 to 4. A total score exceeding 29 indicates possible severe anxiety; over 21 confirms significant anxiety; over 14 confirms anxiety; over 7 indicates possible anxiety; and scores below 6 indicate no anxiety symptoms. The HAMD comprises 17 items [30]. A total score < 7 indicates normal functioning; 7–17 suggests possible depression; 17–24 confirms depression; and > 24 indicates severe depression.

The Montreal Cognitive Assessment (MoCA) evaluates eight cognitive domains: visuospatial processing, executive function, naming, memory, attention, language, abstract reasoning, calculation, and orientation. Patients must be fully conscious and assessed in a quiet environment. The total score ranges from 0 to 30 points, with ≥ 26 indicating normal cognitive function. A score < 26 suggests the presence of cognitive impairment [31].

The Beck Scale for Suicide Ideation (BSI) is used to identify individuals with suicidal tendencies and assess the severity of suicidal thoughts. The scale consists of 19 items, each with three response options (0–2 points), yielding a maximum total score of 38 points. Higher scores indicate greater suicide risk [32].

### MRI data acquisition

Imaging data were acquired on a SIEMENS 3.0 T Trio Tim scanner for all patients. All participants completed MRI scans within 24 ± 2 h following their last esketamine infusion. During the examination, subjects were instructed to lie still on the examination table. Earplugs and an eye mask were worn throughout the procedure to minimize external interference. The head was secured with foam padding to prevent movement, and subjects were instructed to breathe through the nose to minimize interference from mouth breathing on head artifacts. Resting-state functional MRI data (BOLD-fMRI) acquisition: Whole-brain BOLD data were acquired using a Gradient-Recalled Echo-Planar Imaging (GRE-EPI) sequence with the following parameters: TR=2000ms; TE=40ms; flip angle=90º; FOV = 230 mm×230 mm; matrix = 64 × 64; 30 consecutive slices; slice thickness = 4.0 mm; slice spacing = 1.2 mm; scan duration = 8 min 06 s.

### Data preprocessing

The functional images were preprocessed using the Data Processing Assistant for Resting-State fMRI (DPARSF) [33], implemented on the basis of SPM12 and MATLAB 2022b (MathWorks, Natick, MA, USA). To reduce the influence of unstable magnetization at the beginning of scanning, the initial ten volumes were removed. The remaining images then underwent slice-timing correction and motion realignment to improve temporal alignment across slices. Afterward, the data were normalized to the Montreal Neurological Institute (MNI) standard space and resampled to 3 × 3 × 3 mm^3^ voxels, followed by detrending and temporal band-pass filtering at 0.01–0.08 Hz. To further control for motion-related effects, volumes with framewise displacement (FD) exceeding 0.2 mm, together with one volume before and two volumes after, were identified and scrubbed, with the affected time points replaced by spline interpolation.

### INT estimation

The INT for each voxel across the brain was derived from preprocessed resting-state fMRI data, following the approach of a previous study [23]. The procedure involved: (i) computing the autocorrelation function (ACF) of each voxel’s BOLD signal, using time bins equal to the repetition time (TR); (ii) summing the ACF values over the initial positive period, defined from the first lag up to the point immediately before the ACF became non-positive; and (iii) multiplying this sum by the TR to adjust for temporal resolution. Once voxel-wise INT values were obtained, each subject’s whole-brain INT map was smoothed with a Gaussian kernel (FWHM = 6 mm) to improve the signal-to-noise ratio and subsequently Z-transformed to remove individual differences within groups. The resulting map reflects the temporal integration capacity of local neural dynamics for each brain region.

For further analysis, the fMRI Toolbox (GIFT, version 3.0 b) was used. Twenty-five independent components (ICs) were initially extracted using the minimum description length criterion to account for spatial correlations. Group ICA inverse reconstruction was then applied to obtain ICs for each subject group, and the resulting spatial maps and time courses were converted to z-scores [34]. Components were selected for retention based on their maximum spatial correlation with resting-state network (RSN) templates [35]. Eleven RSNs were ultimately identified. IC selection criteria included: (a) peak activity located predominantly in gray matter, (b) minimal overlap with vascular, ventricular, or other artifacts, and (c) temporal signals dominated by low-frequency activity (power below 0.1 Hz relative to 0.15–0.25 Hz). The RSNs identified included the visual network (VN: IC1, IC3, IC13), executive control network (ECN: IC4, IC18), sensorimotor network (SMN: IC5), default mode network (DMN: IC9, IC16, IC20, IC23), and auditory network (AN: IC22).

### Analysis of INT differences between MDD patients and the healthy control group

Generalized linear models (GLMs) were applied to examine between-group regional differences in INT. To better characterize local INT alterations in patients with MDD, cortical areas were further organized based on the Yeo atlas, which defines resting-state functional networks according to cellular structural features. The average INT was then computed for each Yeo-defined network, and case-control differences were evaluated using GLM regression analysis [36]. To control for multiple comparisons across regions, networks, or classes, statistical significance was adjusted using the Benjamini-Hochberg false discovery rate (BH-FDR), with significance set at *p* < 0.05 of cluster level and *p* < 0.001 at voxel level.

### Calculation of regional gene expression levels

Gene expression profiles were obtained from the Allen Human Brain Atlas (AHBA) using data from six postmortem adult human brains, comprising 3,702 distinct tissue samples [37]. Transcriptomic data were processed and mapped to 90 predefined brain regions with the abagen toolbox [38]. The preprocessing pipeline involved reannotating probes, removing probes with low expression intensity, choosing representative probes, assigning samples to brain regions, imputing missing values, normalizing sample-level data, standardizing gene expression values, averaging samples within each region, and selecting genes with consistently stable expression. After preprocessing, 15,633 genes remained for further study. Since only two of the six donors provided samples from the right hemisphere, subsequent analyses were limited to the left hemisphere. As a result, the final dataset consisted of a gene expression matrix covering 45 brain regions and 15,633 genes.This approach follows common practice in imaging-transcriptomic studies and assumes broad left-right symmetry in normative cortical gene expression, although this assumption cannot fully account for potential hemispheric asymmetry and is therefor acknowledged as a limitation.

### Transcription-neuroimaging association analysis

A partial least squares (PLS) regression was performed to examine the association between the expression patterns of 15,633 genes and case-control differences in INT, as indexed by t-values across 45 cortical regions in the left hemisphere [39]. In this model, the predictor matrix consisted of z-score standardized gene expression data (45 regions x 15,633 genes), whereas the response variable was a z-score standardized vector of case-control INT t-values (45 regions x 1). PLS regression extracts latent components representing linear combinations of gene expression variables that maximize the covariance shared with neuroimaging measures. The first latent variable, PLS1, explains the largest proportion of shared covariance, where as subsequent components capture additional, orthogonal gene-neuroimaging covariance patterns.

To determine whether the proportions of variance explained by PLS components were greater than expected by chance, a spatial permutation procedure was applied using a spin test with 10,000 iterations [40]. If none of the PLS components survived spatial permutation correlation, we selected the component with the smallest spatially corrected p value for exploratory downstream analysis, and such results were interpreted as hypothesis-generating rather than confirmatory. In addition, bootstrap resampling was used to estimate the contribution of each gene to the PLS components. Only genes showing significant bootstrap effects after Benjamini-Hochberg false discovery rate correction at *p* < 0.01 were included in subsequent analyses.

To further explore the link between MDD-related gene expression and INT alterations, MDD-associated genes were obtained from the Allen Human Brain Atlas (AHBA; https://help.brain-map.org/display/humanbrain/Documentation). Genes shared between the MDD-related gene set and the full panel of 15,633 genes were identified. The expression levels of these overlapping genes were then correlated with case-control differences in INT across the left hemisphere. Statistical significance was set at *p* < 0.05, with false discovery rate correction applied.

### Enrichment analysis

To examine the functional relevance of the identified genes, we used Metascape, an automated platform for meta-analysis that helps detect pathways and interaction networks shared across multiple gene sets. Gene sets derived from PLS components in patients with MDD were submitted to Metascape to characterize their biological features and to compare them with genes reported in genome-wide association studies (GWAS). Enrichment analysis was conducted using several annotation resources, including Gene Ontology (GO), Reactome, and the Kyoto Encyclopedia of Genes and Genomes (KEGG). Pathways were considered significant at an FDR-adjusted cutoff of 1%. Because no PLS component survived spatial permutation correction at the conventional *p* < 0.05 threshold, the component with the smallest spatially corrected p value (PLS2) was used for exploratory enrichment analysis.

### Locate MDD-related genes to specific cell types

Postmortem cortical datasets were compiled from seven independent single-cell studies. Using a carefully curated collection of gene sets generated from large-scale single-cell profiling of the adult cerebral cortex by Seidlitz et al. [41], seven principal cell populations were defined, including astrocytes, endothelial cells, microglia, excitatory neurons, inhibitory neurons, oligodendrocytes, and oligodendrocyte precursor cells (OPCs). To evaluate the significance of gene overlap for each cell class, permutation testing was conducted, and only overlaps with FDR-adjusted p-values below 0.05 were regarded as statistically significant. The aim was to determine which cell types were spatially associated with MDD-related INT genes. For subsequent cell-type analyses, PLS2 − was chosen because the genes enriched in this component showed both higher loading scores and larger gene counts than those enriched in PLS2+, suggesting greater exploratory biological relevance.

### Associate INT changes with PET neurotransmitter data

To explore the neurochemical mechanisms that may underlie cortical alterations in INT among individuals with MDD, we examined the spatial relationship between regional INT abnormalities and the cortical distribution of neurotransmitter receptors and transporters. For this analysis, we used 39 PET-based maps of receptor and transporter density parcellated according to the AAL90 atlas. These maps were derived from the dataset reported by Hansen et al. (2022) and combine information from multiple tracers and participant subgroups, providing broad coverage of major neuromodulatory systems, including dopaminergic, GABAergic, serotonergic, glutamatergic, and cholinergic pathways [42].

These neurotransmitter systems play essential roles in synaptic signaling, neuronal excitability, and the maintenance of circuit stability, all of which are closely related to the temporal integration processes captured by INT. The PET atlas included receptor and transporter maps for several key targets, such as GABA_A receptors, mGluR5, serotonin receptors 5-HT1a and 5-HT2a, serotonin transporters (5-HTT), dopamine transporters (DAT), and vesicular acetylcholine transporters (VAChT). We then computed the spatial Pearson correlation between the INT alteration map and each receptor/transporter density map. Statistical significance was evaluated using a spin-based permutation test with 10,000 iterations to control for spatial autocorrelation. Multiple-comparison correction was performed using the Benjamini-Hochberg false discovery rate (BH-FDR) procedure.

This multimodal strategy makes it possible to test whether particular neurotransmitter systems, especially those implicated in MDD, contribute to regional susceptibility of INT alterations. In this way, it provides a link between molecular-level neuromodulatory architecture and large-scale temporal dynamics in the human cortex.

### Null model construction

To mitigate the potential influence of spatial autocorrelation in neuroimaging and transcriptomic datasets, we applied a spatial permutation approach based on spherical rotations. This “spin test” maintains the intrinsic spatial organization of the brain while breaking the original spatial correspondence between maps through random rotations on the sphere. A total of 10,000 rotations were conducted to construct a null distribution of correlation coefficients, from which spatially corrected p-values (pspin) were obtained. These values were used to determine whether the associations between INT alterations and gene expression patterns derived from PLS analysis, or PET-based neurotransmitter profiles, exceeded what would be expected by chance due to spatial smoothness. By controlling for spatial dependence, this approach helps ensure that the observed associations are more likely to represent meaningful biological links rather than artifacts driven by anatomical proximity, thereby supporting more robust interpretations of the transcriptional and neurochemical basis of INT alterations in depression.

## Result

### Population, clinical and neurocognitive scales

Summary of demographic and clinical characteristics of the final study participants after exclusion. Table 1 presents the demographic and clinical characteristics of participants. No significant differences were observed between the MDD group and the healthy control group in terms of gender, age, or years of education (41 MDD patients vs. 57 healthy individuals; *p* > 0.05). MDD patients demonstrated higher scores on the HAMA, HAMD, and suicidal ideation scales compared to healthy individuals, while exhibiting lower MoCA total scores. These differences were statistically significant.

**Table 1:** Demographic and clinical data of HC and MDD before treatment

*N*	HC MDD			
	(*n* = 57)	(*n* = 41)	t/z	*P*-value
Gender (Male/female)	17/40	10/31	0.103	0.748
Age (year)^a^	32 ± 11.851	30 ± 11.280	1.063	0.262
Education (year)^b^	12(9,13)	12(9,12)	− 0.277	0.784
HAMA^b^	3(2,4)	23(20,27.5)	-8.459	< 0.001
HAMD^b^	4(3,5)	36(33,40)	-8.463	< 0.001
BSI^b^	0(0,0)	10(8,10)	-8.952	< 0.001
MoCA^b^	28(27,29)	22(19,24.5)	-7.501	< 0.001
MoCA: visual space and execution	5(4,5)	3(2.5,4)	-6.276	< 0.001
MoCA: naming	3(3,3)	3(2,3)	-2.381	0.026
MoCA: delayed memory	4(4,5)	2(1,3)	-6.796	< 0.001
MoCA: attentional function	5(5,6)	5(4,6)	-1.439	0.152
MoCA: language	3(3,3)	3(2,3)	-3.159	< 0.001
MoCA: abstract	2(2,2)	1(1,2)	-4.574	< 0.001
MoCA: orientation	6(6,6)	6(5,6)	-3.645	< 0.001

**Notes**: ^a^ Student, *t*-test; ^b^ Wilcoxon-test. n/a indicates that no statistics

**Abbreviations**: HAMA, Hamilton Anxiety Scale HAMD, Hamilton Depression Scale; BSI, Becker Suicide Ideation Scale༛MoCA, Montreal Cognitive Assessment Scale

### Clinical data of MDD patients before and after treatment

Our study found that 41 patients responded well to esketamine treatment. Compared with baseline, HAMA, HAMD, and BSI scores significantly decreased after treatment; MoCA scores significantly increased after treatment (Table 2).

**Table 2:** Demographic and clinical data before and after MDD treatment

	MDD(*n* = 41)			
	Baseline	Post-treatment	t/z	*P*-value
HAMA^b^	23(20,27.5)	8(5.5,12)	-5.582	< 0.001
HAMD^b^	36(33,40)	15(10,19.5)	-5.581	< 0.001
BSI^b^	10(8,10)	2(2,4)	-5.624	< 0.001
MoCA^b^	22(19,24.5)	27(25,28)	-5.394	< 0.001
MoCA: visual space and execution	3(2.5,4)	4(3,5)	-3.965	< 0.001
MoCA: naming	3(2,3)	3(3,3)	-2.887	0.004
MoCA: delayed memory	2(1,3)	4(3,4)	-5.130	< 0.001
MoCA: attentional function	5(4,6)	6(4.5,6)	-2.394	0.016
MoCA: language	3(2,3)	3(2,3)	-3.124	0.001
MoCA: abstract	1(1,2)	2(1,2)	-3.153	0.002
MoCA: orientation	6(5,6)	6(6,6)	-3.259	< 0.001

Notes: ^a^ Student, *t*-test; ^b^ Wilcoxon-test. n/a indicates that no statistics

Abbreviations: HAMA, Hamilton Anxiety Scale HAMD, Hamilton Depression Scale; BSI, Becker Suicide Ideation Scale༛MoCA, Montreal Cognitive Assessment Scale

### Voxel-wise analysis of intrinsic neural timescale

**Fig. 2:**
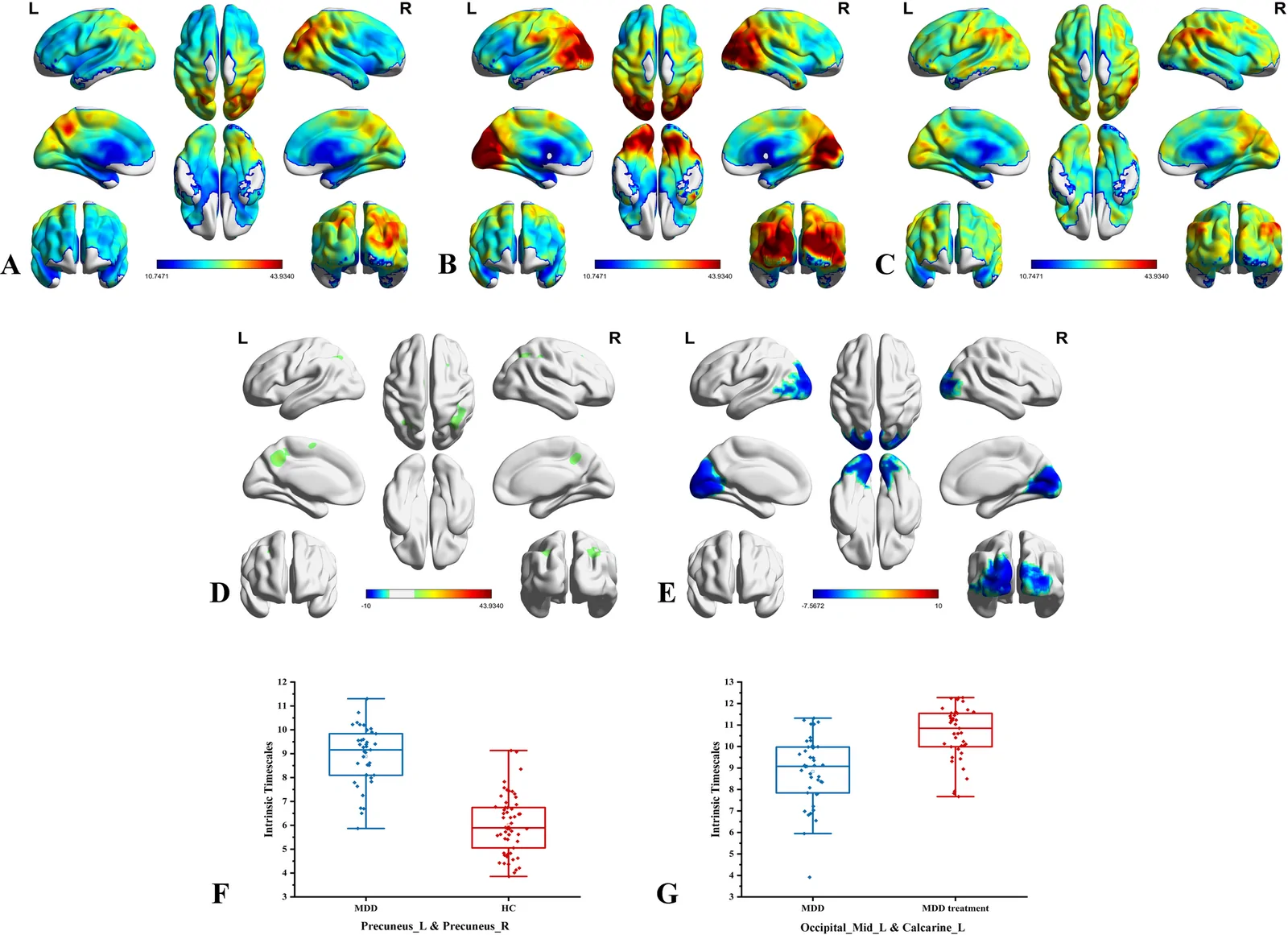
(**A**) Brain INT distribution in MDD patients; (**B**) Brain INT distribution in the treated MDD group; (**C**) Brain INT distribution in the healthy control group; (**D**) Brain INT difference comparison between MDD and HC, showing statistically significant voxels (*p* < 0.05, corrected); (**E**) Brain INT differences between MDD and treated MDD groups, showing statistically significant voxels (*p* < 0.05, corrected); (**F**) Box plots show voxels with significant differences in INT between MDD and HC groups: left Precuneus and Precuneus cortex; (**G**) Box plots show voxels with significant differences in INT between MDD and MDD treatment groups: left middle occipital gyrus and left calcarine cortex; L denotes left hemisphere, R denotes right hemisphere

Our findings revealed significantly elevated INT in the left and right anterior cingulate cortex of untreated MDD patients compared to the healthy control group at both the voxel-level and cluster-level analysis (GRF-corrected, *p* < 0.05) (Fig. 2) (Table 3). Additionally, compared with baseline, post-treatment MDD patients exhibited significantly elevated INT in the left occipital midline region and left calcarine cortex (Fig. 2) (Table 4).

### Network-level analysis of INT

**Fig. 3:**
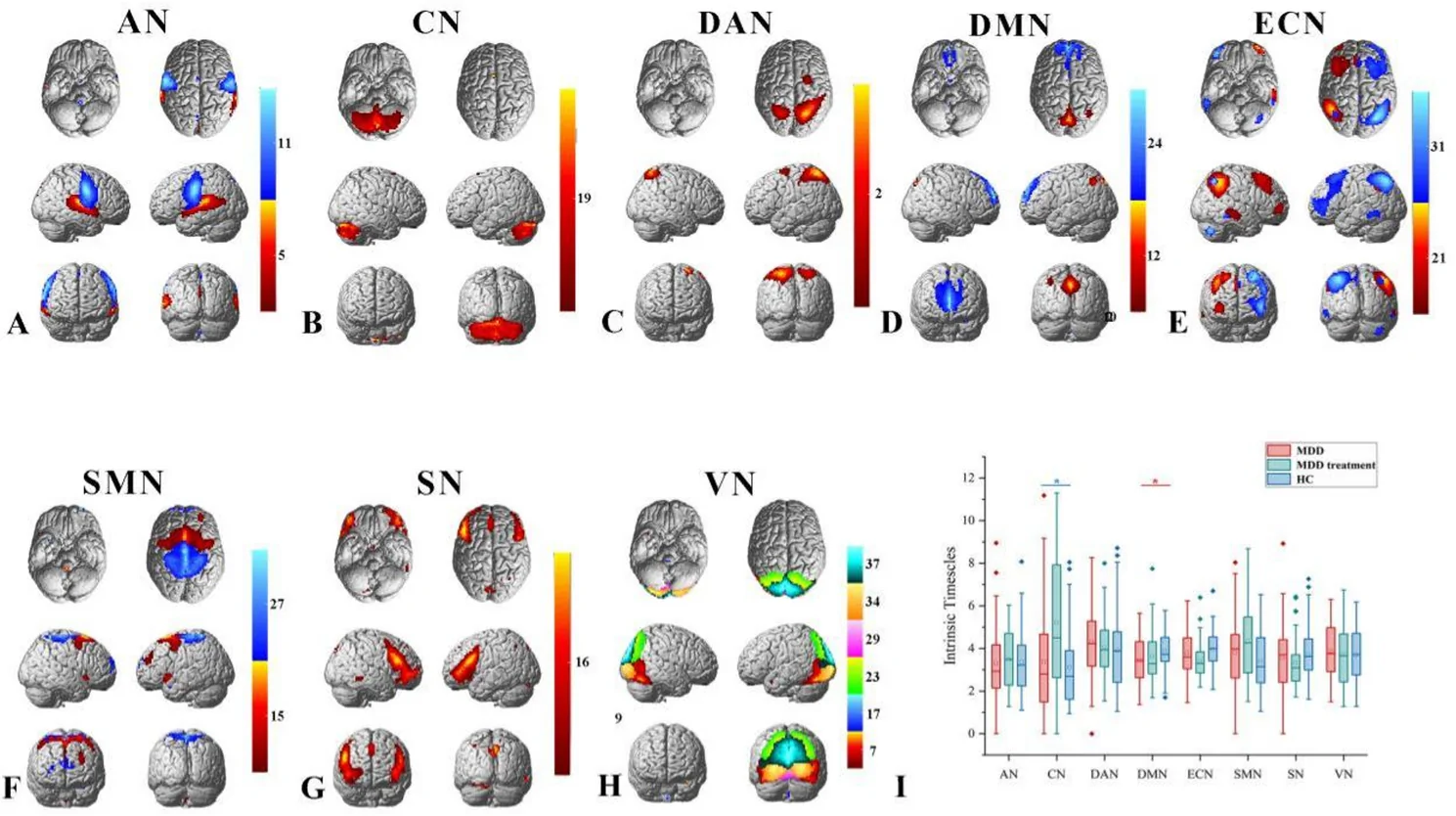
(**A-H**) Resting-state network spatial distribution maps. Auditory Network (AN), Cerebellar Network (CN), Dorsal Attention Network (DAN), Default Mode Network (DMN), Executive Control Network (ECN), Sensorimotor Network (SMN), Salience Network (SN); (**I**) Boxplots illustrate the differences in INT between the MDD, MDD treatment group, and HC groups. Red asterisks indicate brain regions showing significant differences between MDD and HC, specifically the DMN. Blue asterisks denote brain regions exhibiting significant differences between MDD and the MDD treatment group, specifically the CN

To further investigate the distribution of INT within large-scale functional systems, we calculated the average voxel INT values across eight representative brain networks: the Auditory Network (AN), Cerebellar Network (CN), Dorsal Attention Network (DAN), Default Mode Network (DMN), Executive Control Network (ECN), Sensorimotor Network (SMN), Salience Network (SN), and Visual Network (VN). We found that untreated MDD patients exhibited significantly reduced DMN INT compared to the healthy control group. Additionally, the CN network showed a significant increase in INT compared to baseline (Fig. 3).

### Transcriptomic associations with cortical temporal integration alterations in MDD

**Fig. 4:**
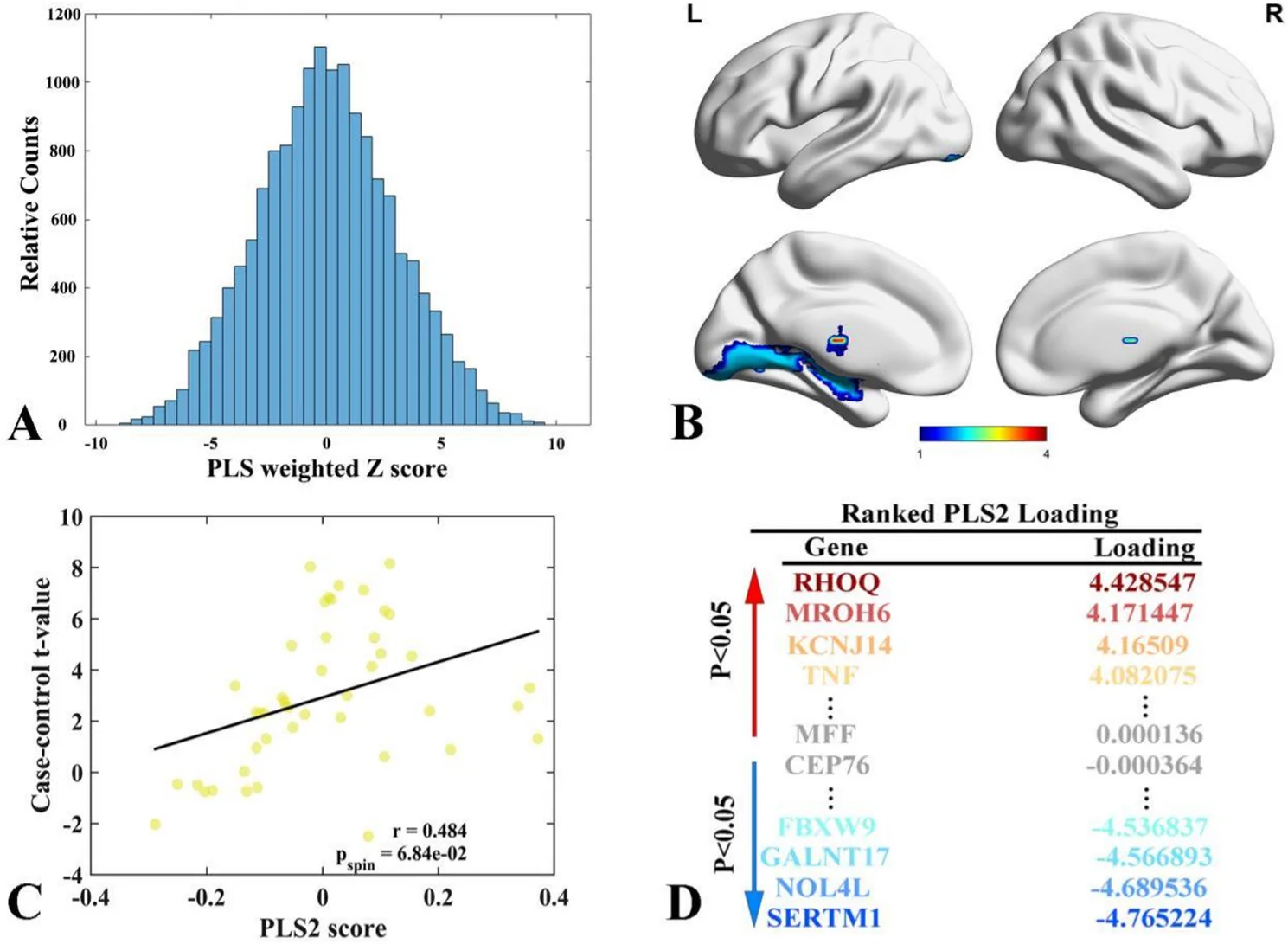
(**A**) Zero distribution plot of PLS2 weighted Z-scores from 10,000 spatial permutation tests, evaluating the relationship between gene expression profiles and imaging-derived PLS2 components; (**B**) Surface projection of PLS2 gene distribution, where colored regions indicate statistically significant PLS2 gene distribution (*p* < 0.05, corrected), in the color map, warmer colors indicate relatively higher distributed gene weights, while cooler colors indicate relatively lower gene weights; (**C**) Spatial correlation exists between PLS2 weights in different regions and t-values from case-control groups (Pearson correlation coefficient: 0.484; Spearman test p-value: 6.84 × 10⁻^2^), indicating a biologically meaningful association between INT alterations and gene expression patterns; (**D**) Gene ranking diagram based on weights: Genes such as RHOQ, MROH6, KCNJ14, and TNF exhibit higher positive weights, while genes including SERTM1, GALNT17, NOL4L, and SERTM1 demonstrate higher negative weights

We performed partial least squares (PLS) regression to investigate the association between cortical INT alterations in MDD and genome-wide spatial gene expression patterns across the entire cerebral cortex. Before presenting the findings related to PLS2, an important caveat must be highlighted: the spatial spin permutation test examining the correlation between PLS2 scores and case–control t-statistic maps of INT differences did not reach the conventional statistical significance threshold. All subsequent spatial correlation analyses and gene enrichment interpretations derived from PLS2 should therefore be regarded as exploratory preliminary observations intended solely for biological hypothesis generation, and the relevant inferences await replication in independent large-scale cohorts.Although the first PLS component (PLS1) accounted for the largest proportion of gene expression variation, it failed to adequately explain the pathological mechanisms of MDD in subsequent analyses. In contrast, the second PLS component (PLS2) captured a substantial proportion of variance (*p* < 0.05, corrected) and revealed biologically meaningful correlations. Notably, the spatial distribution of PLS2 scores differed significantly from the spatially constrained null model, indicating a non-random correspondence between gene expression patterns and PD-associated INT alterations (Fig. 4A).Surface projections of PLS2 scores indicate that enriched genes are predominantly distributed in the anterior brain regions, particularly in the left medial prefrontal cortex and the right temporal and insular margins, exhibiting a significant left-sided distribution (Fig. 4B). PLS2 scores exhibited a positive spatial correlation with case–control t-statistic maps reflecting regional INT differences (*r* = 0.484, p_spin = 0.0684) (Fig. 4C). The spin permutation-derived P value exceeded the conventional threshold of 0.05, indicating that the association failed to pass rigorous significance testing accounting for spatial multiple comparisons. Furthermore, gene loadings ranked by PLS2 revealed distinct gene sets with positive and negative weights (FDR-corrected *p* < 0.05) (Fig. 4D).

### Enrichment analysis

**Fig. 5:**
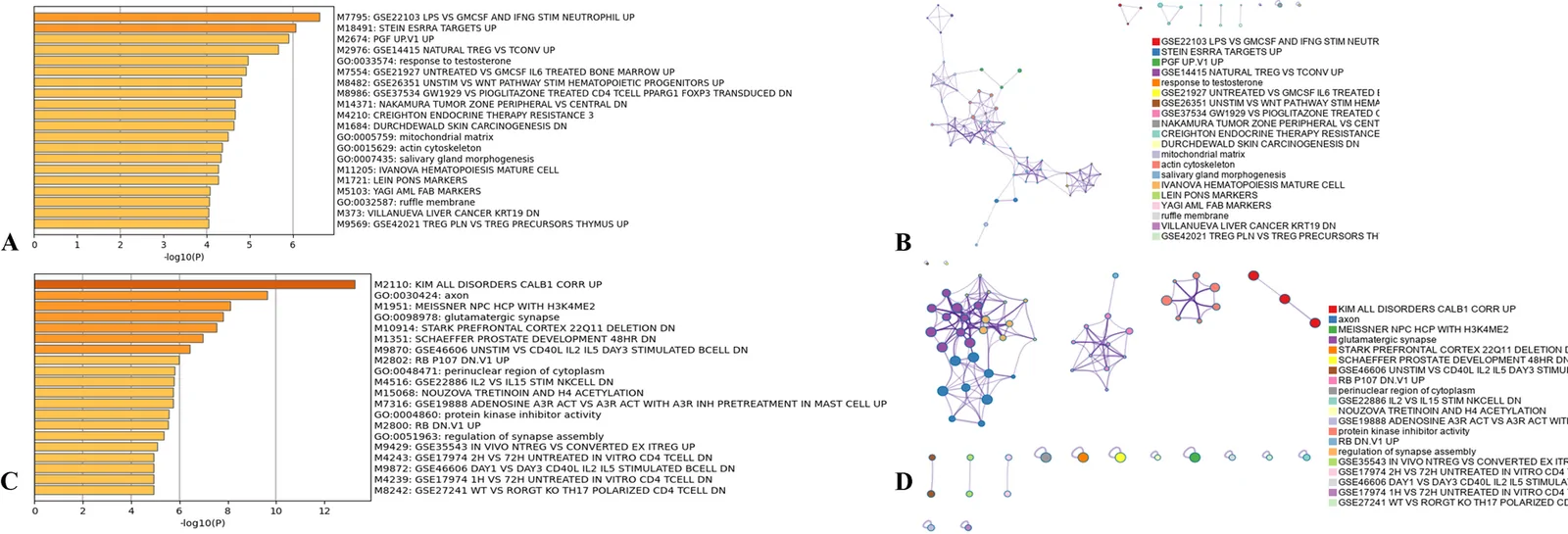
(**A, B**) Functional annotations of genes positively correlated with PLS2 (i.e., exhibiting high positive loadings) are displayed as bar charts (**A**), accompanied by corresponding enrichment networks; (**B**) Bar charts highlight the top-ranked pathways and Gene Ontology (GO) terms based on statistical significance (-log₁₀ p), including processes such as immune response and hormone regulation; Functional annotations of genes negatively correlated with PLS2 are displayed as bar charts (**C**) and network diagrams; (**D**) The most enriched categories include signal transduction and protein kinase inhibition. Each node in the interaction network represents a significantly enriched term or pathway, with color-coded modules reflecting thematic clusters; connected edges indicate functional similarity or shared gene content

To further elucidate the molecular correlates underlying the INT transcriptomic signature observed in MDD patients, we performed gene set enrichment analysis on positively and negatively weighted genes derived from the PLS2 component. Enriched biological processes for positively loaded genes (PLS2+) were primarily associated with immune responses and hormone regulation. We also found pathways related to neutrophils and regulatory T cells to be overrepresented. Additionally, network visualization highlighted the interconnectedness of metabolic pathways, signaling cascades, and neuroinflammatory processes (Fig. 5A and B). In contrast, negatively weighted genes (PLS2-) showed significant enrichment primarily in signal transduction and protein kinase inhibition. These gene sets included components associated with axons, glutamatergic synapses, and synaptic conformation regulation (Fig. 5C and D).

### Transcriptional signatures for canonical brain cell types

**Fig. 6:**
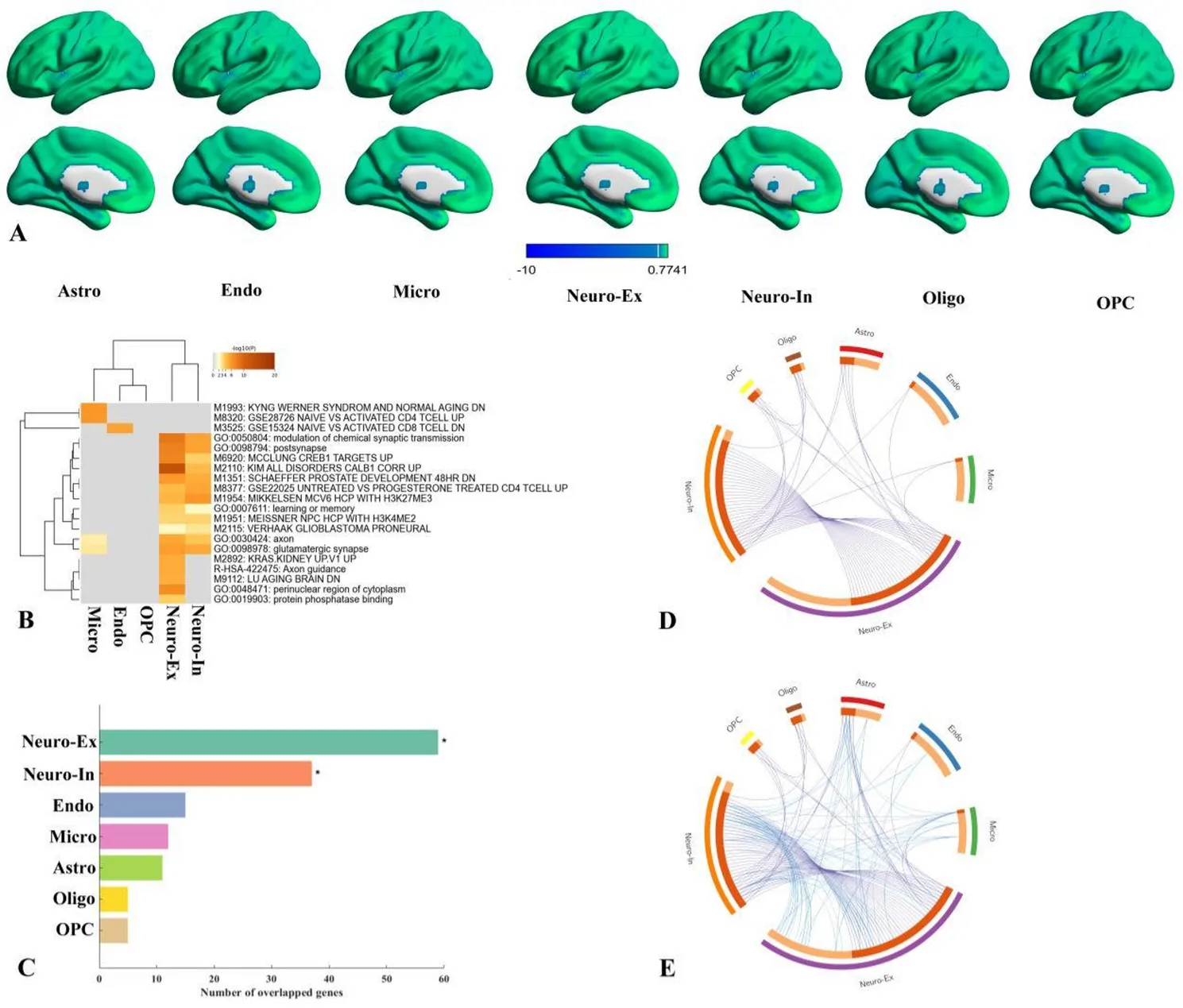
(**A**) Brain projection maps reveal the spatial distribution of INT-associated genes across seven representative brain cell types: astrocytes, endothelial cells, microglia, excitatory neurons, inhibitory neurons, oligodendrocytes, and oligodendrocyte precursor cells. Warmer colors indicate greater magnitude of INT alterations associated with each cell type; (**B**) The heatmap displays the rich Gene Ontology (GO) terms and pathways associated with INT-related genes, highlighting functional differences across distinct cell types; (**C**) The bar chart illustrates the number of overlapping genes between the INT-associated gene set and cell type-specific markers. Regions with significant overlap (FDR-corrected p-value < 0.05) are marked with asterisks; (**D-E**) The pie charts depict the inferred interactions between cell types based on shared INT-associated genes

To further elucidate the underlying cellular mechanisms of MDD-associated INT alterations,

 we mapped disease-related transcriptomic features onto seven representative brain cell types. Spatial projections of cell type-specific expression patterns revealed distinct cortical distributions (Fig. 6A). Enrichment analysis revealed that genes associated with MDD-related changes in INT were significantly enriched in both excitatory (Neuro-Ex) and inhibitory neurons (Neuro-In) (*p* < 0.05, corrected) (Fig. 6C).

Enrichment heatmap results indicate that distinct cell types exhibit highly specific pathway enrichment patterns. Glial cells show significant enrichment in “axons” and “glutamatergic projections.” In contrast, neuronal subtypes, particularly excitatory (Neuro-Ex) and inhibitory (Neuro-In) neurons, were predominantly enriched in pathways associated with synapses and neuronal function, including “axons”, “regulation of chemical synapse transmission”, “glutamatergic synapses”, and “postsynaptic”. Endothelial cell-associated features were specifically enriched in immune pathways. Astrocytes (Astro), oligodendrocytes (Oligo), and oligodendrocyte precursor cells (OPC) did not show significant pathway enrichment (Fig. 6B). To characterize the functional relationships among these distinct cell types, we constructed a circular graph based on shared Gene Ontology (GO) terms within cell type-specific gene sets. Visualization reveals extensive functional overlap among multiple cell types, with particularly strong interconnections observed between neuronal populations (Neuro-Ex and Neuro-In), as indicated by dense purple bands. Notably, neurons-especially excitatory neurons (Neuro-Ex) exhibit widespread connections with nearly all other cell types (Fig. 6D and E).

### Association between INT alterations in PD and neurotransmitter receptor profiles

To assess the contribution of neurotransmitter systems to regional INT alterations in MDD, we evaluated the spatial correlation between regional INT differences and PET-derived density maps across 39 neurotransmitter receptors and transporters. This analysis encompassed a broad range of neuromodulatory systems, including adrenergic transporters, dopaminergic, glutamatergic, GABAergic, serotonergic, and cholinergic systems.

**Fig. 7:**
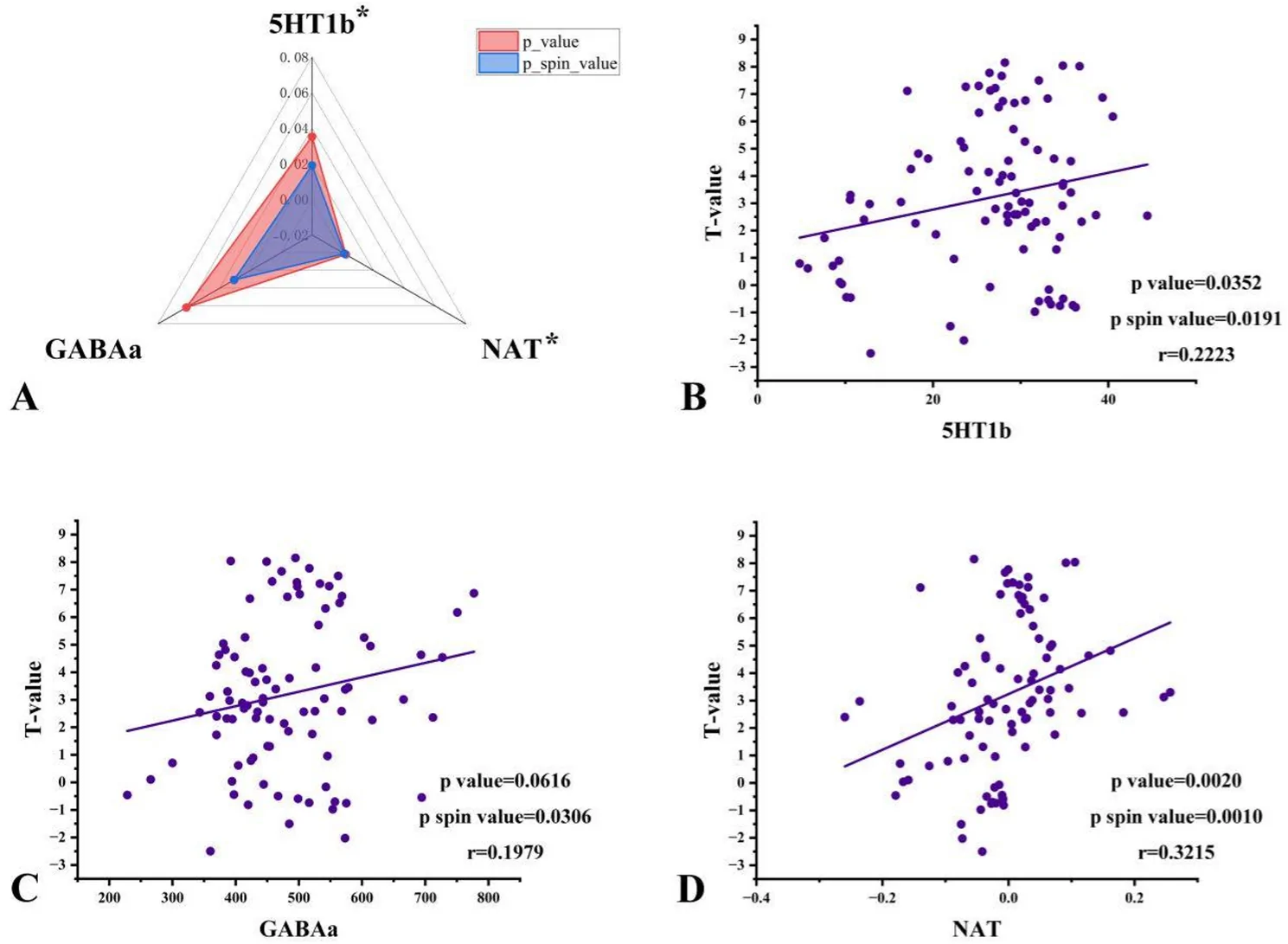
(**A**) The central radar chart displays the correlation strength (T-value) between INT morphological similarity patterns and the density of three neurotransmitter receptors (5HT1b, GABAa, and NAT). The two curves represent the uncorrected p-value for INT (red) and the p-value corrected for rotation tests (blue); (**B-D**)The scatter plot depicts the linear correlation between receptor density and INT T values, annotated with the regression line, Pearson correlation coefficient, uncorrected p-value, and p-value corrected by rotation test. Receptors showing statistically significant enrichment (FDR < 0.05) are marked with an asterisk (*)

The initial correlations revealed several receptor types that showed nominal associations with the spatial patterns of INT alterations in MDD. However, after correcting spatial autocorrelation using spin tests, only the 5HT1b receptor (*r* = 0.2223, *p* = 0.0352, p_spin = 0.0191) and NAT (*r* = 0.3215, *p* = 0.002, p_spin = 0.001) remained significantly correlated with cortical INT changes. It is noteworthy that although GABAa receptors showed potential correlations, these associations did not remain statistically significant after spatial correction (Fig. 7).

**Table 3:** Voxel differences between MDD and the healthy control group

Conditions	Brain Regions	Cluster Sizes (voxels)	MNI Coordination			t-Score of Peak Voxels
			X	Y	Z	
MDD > HC	Precuneus_L / R	806	-9	-51	45	14.0493

Abbreviations: MDD, Major depressive disorder; HC, healthy control; MNI, Montreal Neurological Institute

**Table 4:** Voxel differences after MDD treatment

Conditions	Brain Regions	Cluster Sizes (voxels)	MNI Coordination			t-Score of Peak Voxels
			X	Y	Z	
MDD < MDD treatment	Occipital_Mid_L / Calcarine_L	542	0	-87	6	-7.5672

Abbreviations: MDD, Major depressive disorder; HC, healthy control; MNI, Montreal Neurological Institute

## Discussion

Our study revealed the following findings. First, voxel-wise analysis demonstrated significantly elevated INT levels in the left and right precuneus of patients with MDD compared with healthy controls (GRF-corrected, *p* < 0.05). In addition, post-treatment INT levels in the left occipital midline region and left calcarine cortex of patients with MDD were significantly increased relative to baseline. Second, after false discovery rate (FDR) correction at the large-scale network level across eight typical networks, the DMN exhibited a significant reduction in INT between the MDD and HC groups, whereas the CN showed a significant increase in INT after treatment. Third, PLS regression analysis identified PLS2 as the primary component associated with INT alterations. Enrichment analysis of PLS2 + genes revealed key pathways related to the immune response and hormone regulation, neutrophils, and regulatory T cells (*r* = 0.484, p-spin = 0.0684). PLS2 − genes were associated with signal transduction and protein kinase pathways, including axons, glutamatergic synapses, and regulatory synapses. Fourth, cell-type enrichment analysis revealed that PLS2 was significantly associated with excitatory and inhibitory neurons (FDR < 0.05), indicating that major neuronal types contribute to INT susceptibility. Finally, among the 39 PET-derived neurotransmitter receptor maps, 5-HT1b (*r* = 0.2223, *p* = 0.0352) and NAT (*r* = 0.3215, *p* = 0.0020) were significantly correlated with changes in INT.

Our results indicate that patients with MDD exhibit significantly elevated INT in both precunei compared with healthy controls. The precuneus is a heterogeneous brain region involved in diverse cognitive functions, including visual processing, mental imagery, and episodic information [43]. Previous studies have linked localized precuneus dysfunction to depression. Zhang et al. found that increased spontaneous neural activity in the precuneus may contribute to cognitive and sensory dysfunction, particularly in the early stages of depression [44]. Abnormal functional connectivity has also been identified between the precuneus and several other brain regions. Fan et al. found that, in patients with MDD and childhood trauma, reduced functional connectivity between the right precuneus and amygdala was significantly associated with diminished physical and social pleasure [45]. Consistent with these studies, we observed significantly elevated INT values in the bilateral precuneus of patients with MDD. This finding indicates prolonged temporal persistence of neural activity and extended information integration within the precuneus, which may contribute to the persistent rumination frequently observed in individuals with depression. The precuneus is a critical node of the DMN [46]. At the large-scale network level, we observed a notable decrease in INT within the DMN of patients with MDD. This result is consistent with previous studies. Zhang et al. found that adolescents with MDD exhibited significantly reduced INT in the DMN across multiple brain regions, including the prefrontal cortex, angular gyrus, middle occipital gyrus, and cerebellum. This reduction in INT within higher-order cortical areas was significantly correlated with disease severity [47]. Reduced DMN integrity indicates that a shortened temporal integration window may lead to inefficient processing of self-related information, potentially explaining the persistence and ineffectiveness of rumination in patients with MDD [48]. We observed globally reduced INT alongside focally elevated INT within the DMN, suggesting heterogeneous temporal abnormalities across discrete DMN subregions rather than uniform global temporal compression. We hypothesize that this network-wide temporal dysregulation may partially account for key clinical phenotypes of MDD, including negative rumination and anhedonia [49].

Compared with pre-treatment levels, INT increased significantly in the left occipital mid-region and left calcarine cortex in patients with MDD after treatment. The left occipital insular region and left calcarine cortex both belong to the primary visual cortex [50]and participate in integrating information into visual working memory [51]. Previous studies of untreated MDD have identified functional alterations in the primary visual cortex [52]. Ketamine treatment may modulate emotional disturbances in MDD by altering neural activity in the occipital lobe. A study of patients with treatment-resistant depression found that ketamine therapy increased glucose metabolism in the bilateral occipital lobes, left hippocampus, and left inferior parietal cortex. These localized metabolic changes were associated with rapid antidepressant effects [53]. Another study found that enhanced functional connectivity between the occipital-parietal and cingulate cortices after ketamine treatment in patients with MDD was associated with rapid antidepressant effects [18]. Consistent with these findings, our results demonstrated altered INT within the primary visual cortex. We speculate that pharmacological treatments may ameliorate impaired visual information processing in patients with MDD. At the brain network level, we did not observe normalization of the DMN; instead, we found increased INT in the CN. Previous studies have provided evidence that ketamine modulates mood disorders by regulating cerebellar function [54]. Loureiro et al. found that ketamine modulated functional connectivity between the cerebellum and large-scale cortical networks, including the frontoparietal and salience networks [55]. Consistent with these findings, we identified prolonged synchronous coupling of intracerebellar neurons. Temporal remodeling within the cerebellar network may partially mediate DMN hypofunction and provides evidence for recovery patterns of temporal integration deficits in MDD. We cannot fully attribute the observed INT alterations to the pharmacological effects of esketamine. Previous functional neuroimaging studies have reported that sertraline alleviates depressive symptoms by modulating brain function. However, robust therapeutic alterations induced by most SSRIs typically require eight weeks or longer to emerge, likely because of their mechanisms of action [56]. In contrast, esketamine exerts rapid antidepressant effects through glutamatergic signaling pathways, as documented in previous investigations. Accordingly, the rapid antidepressant response and concurrent cerebral functional changes observed within the two-week window in the present study are more likely attributable to esketamine administration [57].

Our functional enrichment analysis revealed disease-specific shifts in biological processes associated with gene sets linked to PLS2-mediated INT alterations. This exploratory analysis highlighted functional themes related to immune responses, hormonal regulation, neutrophils, and regulatory T cells. Abnormal immune activation and hormonal dysregulation are key biological mechanisms underlying the onset and progression of depression. Immune activation leads to the release of pro-inflammatory cytokines (IL-1γ, IL-6, tumor necrosis factor) by fibroblasts, endothelial cells, and epithelial cells in the periphery [58]. These cytokines transmit inflammatory signals to the brain through vulnerable regions of the blood-brain barrier, activating the hypothalamic-pituitary-adrenal axis and intensifying the brain’s stress response. Hormonal regulation, particularly through the hypothalamic-pituitary-adrenal axis, releases adrenocorticotropic hormones that regulate the stress hormone cortisol [59]. Elevated cortisol levels suppress receptors in the hippocampus and hypothalamus, leading to functional imbalance in these structures. Persistently high cortisol levels also promote inflammatory responses [60]. Increasing evidence indicates that elevated immune cell levels can induce inflammatory states and thereby contribute to depression progression. Previous studies have shown that peripheral circulating immune cells may induce immune dysfunction by coordinating immune responses and producing cytokines and immunoglobulins [61], involving neutrophils and T cells [62]. Consistent with trends reported in previous studies, our analyses revealed associations between INT metrics and biological processes involving immune responses, hormonal regulatory pathways, and multiple immune cell populations. Based on these observations, we propose the tentative hypothesis that aberrant immune-neuroregulatory crosstalk may partially mediate temporally dysregulated and imbalanced signaling across select cortical regions in depressive states.

In addition, our exploratory analyses revealed associations among INT metrics, signal transduction, and protein kinase pathways within the negative-weighted gene set (PLS2−). These pathways included components related to axonal function, glutamatergic synapses, and synaptic conformation regulation. Previous studies have demonstrated that the glutamatergic system is implicated in the pathophysiology of mood disorders [63]and participates in emotional regulation by modulating the balance between inhibitory and excitatory neurotransmitters. Evidence from post-mortem studies also indicates that emotional disorders are associated with the expression of glutamatergic genes [64], particularly GRIA3, GRIK2, GRIK4, and GRM7 [65]. Consistent with these findings, our results indicate an association between INT metrics and the expression of glutamatergic synapse-related genes. Given that these enrichment signatures lack robust statistical support, we propose a tentative hypothesis requiring further validation: proteins encoded by glutamatergic synaptic genes may participate in synaptic plasticity, which could partially contribute to neuronal integration of synaptic inputs and sustained neural activity [66].

Enrichment analyses derived from PLS2 served as preliminary exploratory evidence in the present study and showed that INT-correlated genes were prominently enriched in patterns linked to excitatory and inhibitory neurons. Excitatory neurons are primarily associated with pathways related to synaptic signaling, axonal development, and neuronal projection targeting and are crucial for maintaining long-distance communication within the brain [67]. Inhibitory neurons are enriched in genes associated with synaptic organization, local inhibition, and circuit regulation and play a crucial role in maintaining cortical computation [68]. The functional balance between excitatory and inhibitory neurons underpins brain function, and its disruption is considered one of the root causes of neurological disorders [69]. Zhu et al. found that, in patients with depression, changes in structural functional connectivity coupling between the default mode network and frontoparietal network are associated with specific gene expression patterns. These enriched genes were primarily related to excitatory neurons, inhibitory neurons, cellular metabolism, and synaptic function [70]. Liu et al. found that, following electroconvulsive therapy in patients with MDD, increased gray matter volume in the motor, prefrontal, and insular cortices was associated with specific functional pathways and cell types; PLS1 was enriched in excitatory and inhibitory neurons [71]. Another study integrated single-cell and bulk-tissue quantitative trait locus data, revealing that disease-causing genes for MDD exhibited specificity across different brain cell types, including excitatory neurons, astrocytes, and oligodendrocytes [70]. Drawing on prior studies and the exploratory molecular imaging coupling signatures observed in the present work, we propose a tentative hypothesis requiring further validation: imbalance between excitatory and inhibitory neurons may correlate with region-specific INT alterations, and such dyshomeostasis could partially mediate impaired information-processing efficiency in patients with MDD.

Additionally, we observed significant spatial correlations between changes in INT and the distribution patterns of PET-derived neurotransmitter receptors, specifically 5-HT1b and NAT receptors. The 5-HT1b receptor may represent a potential therapeutic target for depression [72], as it not only reduces synaptic 5-HT release through negative feedback mechanisms but also modulates the release of other neurotransmitters (acetylcholine, dopamine) [73]. Previous imaging studies have also found that reduced 5-HT1b receptor binding in the raphe nuclei and increased 5-HT1b receptor binding in certain occipital and temporal cortical regions are associated with improved mood [74]. Noradrenergic receptors originate in the locus coeruleus and project to multiple regions of the forebrain. Their functions primarily involve regulating arousal, stress responses, and memory consolidation [75]. Chen et al. found that following treatment with noradrenergic receptor-targeted medications, changes in functional connectivity patterns between the locus coeruleus and the whole brain in patients with major depressive disorder (MDD) corresponded to brain regions where norepinephrine transporters are distributed [76]. Our findings indicate that brain regions with altered INT levels in patients with MDD demonstrate higher densities of 5-HT1b and NAT receptors, suggesting that serotonergic and noradrenergic systems may be linked to temporal integration abnormalities in MDD.

## Limitations

Several limitations should be acknowledged. First, all patients with MDD received combined esketamine and sertraline treatment throughout the 2-week intervention, precluding the differentiation of the independent and synergistic effects of the two agents. Second, our imaging-transcriptomic analyses were based on the Allen Human Brain Atlas (AHBA), which provides gene expression data primarily from the left cerebral hemisphere. Because the INT abnormalities identified by resting-state fMRI involved bilateral cortical regions, cross-hemisphere mapping was required. Although this approach is widely adopted and supported by the overall hemispheric symmetry of cortical gene expression, subtle hemisphere-specific expression patterns cannot be excluded and may have affected the robustness of the molecular-imaging associations. Third, the rapid synaptic effects of esketamine are time-dependent, and variability in MRI acquisition intervals may have introduced additional confounding. Finally, the AHBA contains transcriptomic data from healthy donors rather than patients with MDD. Given the widespread transcriptional and epigenetic alterations associated with depression, linking pathological imaging changes to a healthy molecular reference may introduce biological bias. Future studies incorporating depression-specific transcriptomic datasets and independent treatment arms with standardized imaging schedules are warranted to validate and extend these findings.

## Conclusion

Our preliminary data indicate that the combination of esketamine and sertraline demonstrates significant antidepressant effects, markedly improving symptoms of MDD. At the voxel level, we found that emotional disturbances and recovery in MDD may be associated with changes in the INT network within specific cortical regions. At the brain network level, MDD patients exhibit alterations in the DMN network’s INT activity. The rapid recovery of emotional disturbances following treatment in MDD patients may be related to compensatory changes in the CN network. Furthermore, transcriptomic analysis revealed distinct molecular signatures underlying INT-associated gene alterations. PLS2 + genes were associated with immune responses, hormonal regulation, and immune cells, while PLS2- genes were linked to signal transduction and protein kinases, and showed significant correlations with excitatory and inhibitory neuronal cell types. Notably, alterations in INT spatially correlate with cortical 5-HT1b receptor and NAT receptor densities, highlighting the critical role of neurotransmitters in regulating temporal integration during MDD. Collectively, these findings underscore the necessity of integrating neurodynamics, gene expression, and receptor localization to enhance our understanding of regional cortical vulnerability in MDD.

## Data Availability

The data that support the findings of this study are available from the First Affiliated Hospital of Nanchang University but restrictions apply to the availability of these data, which were used under license for the current study, and so are not publicly available. Data are however available from the corresponding author upon reasonable request and with permission of the First Affiliated Hospital of Nanchang University.
